# Fertilization reduces root architecture plasticity in *Ulmus pumila* used for afforesting Mongolian semi-arid steppe

**DOI:** 10.3389/fpls.2022.878299

**Published:** 2022-07-19

**Authors:** Antonio Montagnoli, Bruno Lasserre, Mattia Terzaghi, Ser-Oddamba Byambadorj, Batkhuu Nyam-Osor, Gabriella Stefania Scippa, Donato Chiatante

**Affiliations:** ^1^Laboratory of Environmental and Applied Botany, Department of Biotechnology and Life Science, University of Insubria, Varese, Italy; ^2^Department of Biosciences and Territory, University of Molise, Pesche, Italy; ^3^Department of Chemistry and Biology ‘A. Zambelli’, University of Salerno, Fisciano, Italy; ^4^Laboratory of Forest Genetics and Ecophysiology, School of Engineering and Applied Sciences, National University of Mongolia, Ulaanbaatar, Mongolia; ^5^Laboratory of Silviculture, College of Agriculture and Life Science, Chungnam National University, Deajeon, South Korea

**Keywords:** Siberian elm, land degradation, forest shelterbelt, forest restoration, functional root traits, root system architecture, root topology, tree anchorage

## Abstract

In this study, we assessed the functional and architectural traits in the coarse roots of *Ulmus pumila* trees, which are used for afforesting the semi-arid steppe of Mongolia. Tree growth was supported by different watering regimes (no watering, 2, 4, and 8 L h^−1^) and by two types of soil fertilization (NPK and compost). In July, 2019, for each of these treatments six trees, outplanted in 2011 as 2-year-old seedlings from a container nursery, were randomly selected, excavated by hand, and digitized. The build-up of root length correlated positively with increasing levels of watering for both soil depths analyzed. The application of fertilizers led to root growth suppression resulting in a general reduction of root length in a lowered rooting depth. When root system characteristics were analyzed in relation to wind direction, unfertilized trees showed higher root diameter values in both soil layers of leeward quadrants, likely a response to mechanical forces to improve stability. On the contrary, fertilized trees did not show differences in root diameter among the different quadrants underscoring a strong reduction in root plasticity with a lack of morpho-architectural response to the mechanical forces generated by the two prevailing winds. Finally, the root branching density, another important trait for fast dissipation of mechanical forces, was significantly reduced by the fertilization, independently of the quadrants and watering regime. Our results suggest that knowledge of the root response to the afforestation techniques applied in the semi-arid steppe of Mongolia is a necessary step for revealing the susceptibility of this forest shelterbelt to the exacerbating environmental conditions caused by climate change and, thus, to the development of a sustainable and successful strategy to restore degraded lands.

## Introduction

In recent studies, afforestation has been recognized as one of the major climate change mitigation solutions (Doelman et al., 2020). Various afforestation programs have been implemented worldwide as an effective measure toward the sustainable provision of ecosystem services, such as carbon sequestration, soil erosion control, and water quality improvement (Xiao et al., 2019). The scientific community has highlighted that together with the demands emerging from a changing society (Montagnoli, 2018), afforestation activities must consider the influence of anthropogenic alterations on global ecosystems (Foley et al., 2005; Kareiva et al., 2007; Ellis et al., 2013) since sites requiring restoration will become increasingly harsher (Oliet and Jacobs, 2012; Stanturf et al., 2014).

Mongolian arid and semi-arid lands are highly degraded and prone to desertification (Tsogtbaatar, 2004, 2009). In 2008, to counteract soil degradation and promote agroforestry activities, the Mongolian and South Korean governments jointly promoted a forested shelterbelt named the *Green Belt Project* (Lee and Ahn, 2016; Byambadorj et al., 2021a,b,c; Nyam-Osor et al., 2021). The establishment of a new tree plantation in these lands represents a difficult task, especially when extreme weather events, such as cold spells, heat waves, drought, and windstorms are expected to occur more frequently or with greater severity due to climate change (Meehl et al., 2000; Harris et al., 2006; Allen, 2009; Allen et al., 2010). The interplay of these adverse environmental factors has the potential to make afforestation and reforestation efforts unsuccessful (Choi, 2004; Cao, 2008; Wang et al., 2010). In particular, windstorms are among the primary causes of destruction in forests (McCarthy et al., 2010; Yang et al., 2014). For example, in European lands, during the past half-century, it has been observed that the increasing number and intensity of storms (Gregow et al., 2017) caused severe damage to forest trees and a great loss of timber (Stokes, 2002; Schelhaas et al., 2003; Schelhaas, 2008; Danjon et al., 2013).

One of the main types of forest tree damage is anchorage failure. Root anchorage has to be especially efficient in forest trees, since they are particularly vulnerable to strong winds due to their tall stems, as they grow to enhance light availability against competitors (Anten et al., 2005). To resist mechanical stresses deriving from the wind force acting on the tree canopy but also from the above-ground organs’ weight, trees need to transfer external forces down into the soil through the roots as rapidly and efficiently as possible (Danjon et al., 2013; Dumroese et al., 2019; Montagnoli et al., 2020). For these reasons, structural modifications of the spatial configurations of the root system, referred to as root system architecture (RSA), that lead to a differentiated rooting depth and spatial placement of the root axes represent the available and possible routes along which the mechanical forces are smoothly and rapidly dissipated into the soil (Stokes and Mattheck, 1996; Danjon et al., 2005; Dupuy et al., 2005, 2007). The efficiency by which this process occurs has a marked effect on achieving mechanical strength and improving the resistance of forest trees to overturning in windy climates (Nicoll and Ray, 1996; Stokes et al., 1996; Dumroese et al., 2019; Montagnoli et al., 2020). Thus, under stressed conditions, the stability of trees can increase significantly, resulting in a large and selective acclimation to prevailing winds (Danjon et al., 2005), by modifying the circular distribution of shallow roots in relation to the prevailing wind azimuth (Nicoll et al., 1995; Quine and Gardiner, 2007; Dumroese et al., 2019; Montagnoli et al., 2020). Concerning this topic, Dumroese et al. (2019) have recently shown that in Ponderosa pine shallow root length and volume were highest both downslope and windward, while at greater depths they were the highest upslope and leeward.

Tree stability may also be enhanced by a highly branched root system since the high density of roots per unit area of soil enables mechanical forces to be transferred rapidly to the soil (Stokes and Mattheck, 1996). In addition, as bending strength is proportional to the root diameter to the third power, a lower tapering degree (i.e., higher mean root diameter) would help resist loading on the root system (Stokes and Mattheck, 1996). Researchers showed that a significant role in tree stability and soil reinforcement must be accounted for roots with large diameters (Kodrík and Kodrík, 2002; Cohen and Schwarz, 2017). Thus, roots of larger trees can bear higher tensile and compressive forces owing to higher root densities and more roots of larger diameters. Furthermore, Coutts (1983, 1986) and Stokes and Mattheck (1996) showed that the windward lateral roots and related architectural traits (length, diameter, and branching pattern), held in tension were the most important component of root anchorage, followed by the root/soil plate weight, soil cohesion, and the bending strength of leeward roots. In addition, soil type and rooting depth also influence the resistance to uprooting as well as the mechanisms occurring during tree failure (Dupuy et al., 2005). Indeed, through a numerical investigation, Dupuy et al. (2005) showed that in sandy soils the field of plastic strain on the windward side was much more developed. The dissymmetry of the plastic strain corresponded to the uprooting mechanisms measured in the field by other authors (Stokes, 1999; Mickovski and Ennos, 2002; Cucchi et al., 2004). Later, Dupuy et al. (2007) also highlighted that the rooting depth was a determinant parameter in sandy-like soil with a positive relationship between the number of vertical roots embedded in the soil, the soil/root cohesion, and the anchorage strength.

Besides in response to mechanical forces, and in addition to genetically determined developmental programs, RSA is regulated by signals from the surrounding soil environment, such as water and nutrient availability (Montagnoli et al., 2019; Karlova et al., 2021). Root plasticity is the phenomenon that led to the alteration of the root phenotype in response to the environment for optimizing the uptake of edaphic resources. Indeed, roots have evolutionarily acquired tremendous plasticity regarding the geometric arrangement of an individual root and the three-dimensional organization of the entire system within the soil (Sultan, 2000; Fromm, 2019). A plant’s ability to explore the soil and compete for soil resources is largely dependent on the architecture of its root system (Lynch, 1995). The continuous fluctuation in space and time of water and nutrients may induce the root system to respond through plastic morphological adaptation (Hodge, 2003; Hwang et al., 2007; Giehl et al., 2013), thereby determining the volume of soil explored, and significantly impacting efficiency in acquiring resources (Morris et al., 2017). Root traits, such as length, branching characteristics, and diameter have been quite often related to the foraging strategies. There is scientific consensus that root branching is subject to genetic control and influenced by biotic and abiotic factors (Duque and Villordon, 2019). Lynch (2019) highlighted how reduced root production (i.e., lateral root branching density) is beneficial for N capture by reducing competition among root axes for resources. Also, Rewald et al. (2011) found that water flux was highly related to the root order while other authors related the change in water and nutrient availability to plastic modification of the diameter and length of the root population (Montagnoli et al., 2018, 2019). Therefore, understanding RSA plasticity in relation to external influencing factors (e.g., mechanical forces, water, and nutrient availability) can give insights into the adaptability of plant species, their development, and the deriving potential tree stability, especially in an environment characterized by harsh conditions and extensive soil degradation (Karlova et al., 2021).

This is particularly true for trees growing at afforestation sites of the Mongolian steppe, which may use different strategies to adapt their roots to variations in seasonal climates and are subjected to different management techniques such as fertilization type and watering regimes (Ma et al., 2018). Indeed, since soil characteristics of the afforested area might not be adequate for a tree cover, the root developmental plasticity has been a major determinant for the success of land plants (Wilkinson, 2000; Hodge, 2004; Morris et al., 2017) and the stability of trees which once fully grown could be in danger of failing. In a recent study, where the entire root system, once digitized, was dissected across different diameter classes, oven-dried, and weighed, Nyam-Osor et al. (2021) showed that when fertilization was coupled to watering regimes it suppressed root biomass in all root categories considered while increasing only the level of watering directly increased root biomass.

The tree species analyzed in this study, *Ulmus pumila* L. (Siberian elm), has been used previously for afforestation projects due to its great adaptability to grow on soils with limited water and nutrient availability (Moore, 2003; Engelbrecht et al., 2005). This characteristic was confirmed by the eco-morpho-physiological traits measured after 10 years of growth, which showed that the low productivity found for *U. pumila* trees was related to a slow growth habit and reduced water use (Cho et al., 2019; Byambadorj et al., 2021a,b,c; Nyam-Osor et al., 2021). Furthermore, it has been demonstrated that root biomass development, stem height, and collar diameter increments observed in *U. pumila* trees were less dependent on watering regimes but more negatively influenced by the application of fertilizers (Nyam-Osor et al., 2021). Finally, *U. pumila* trees showed high survival rates even without irrigation.

For the present study, we hypothesized that the root biomass suppression reported in Nyam-Osor et al. (2021) due to the application of fertilizers, would also negatively affect the root architectural plasticity reducing the root length, the mean diameter of the root population, and the branching density. In particular, we expected that the use of fertilizers would result in a lower rooting depth and a reduced root morphological response to prevailing wind related-sectors. To test our hypotheses, coarse roots’ length, diameter, and branching density were analyzed with both spatial distribution (vertical and horizontal) and management techniques. Our objective was to understand the root growth modulation in response to environmental conditions, and the influence of the adopted management techniques on this modulation. Indeed, although of fundamental importance for the afforestation programs root functional traits are scarcely studied. Our approach would enable scientists and practitioners to predict the response of afforestation stands to more severe, climate change-induced storms, and to carefully select both plant material and management techniques to adequately support the survival and rapid development of planted trees to ensure the long-term success of restoration interventions.

## Materials and methods

### Site description

The experimental site is located in Lun soum (Tuv province, Mongolia; 47°52′15.43′N, 105°10′46.4′E) on the right bank of the Tuul River, 135 km west of Ulaanbaatar at an elevation of 1,130 m a.s.l. The site extends 2 ha within the forest nursery of the South Korea-Mongolia joint *Green Belt* plantation project in the Middle Khalkha dry steppe region (Ulziykhutag, 1989), which has been greatly degraded by intense livestock grazing.

The area is fairly flat with a soil type classified as Kastanozems (Loamic), which is more than 1 m deep, immature, and lacking horizontal development. The hardness of the topsoil is 4.5 kg cm^−2^, while that of the subsoil is 1.7 kg cm^−2^, as the topsoil is drier than the subsoil (IUSS Working Group WRB, 2015; Batkhishig, 2016).

In regard to chemical characteristics, the soil pH average is 7.37 with similar values for the whole soil depth intervals analyzed. The soil carbon percentage is absent (0%) for the whole soil depth analyzed with the only exception of the deepest soil layer (90–10 cm) where was 0.95%. The organic matter (%) is around 0.8 for the whole soil depth analyzed with the lowest values (0.5 and 0.2) measured in the deepest soil layers (80–90 and 90–100 cm). Nitrate-nitrogen content (mg kg^−1^) values along the soil profile have values around the average of 7.2. Electrical conductivity (dS m^−1^) has an average value of 0.04 increasing progressively from the upper to the lower soil depth analyzed. Phosphorus pentoxide (P_2_O_5_; mg/100 g) has an average value of 1.16 with values along the soil depth spamming from 0.68 (30–40 cm) to 1.77 (40–50 cm). Potassium oxide (K_2_O; mg/100 g) has an average value of 6.3 with the highest values in the upper 30 cm of soil (from 6.7 to 10.2) and then 5.5 for the remaining soil depth (Table 1).

**Table 1:** Profile characteristics of the experimental site Kastanozems (Loamic) soil type.

Depth (cm)	рН	Carbon (%)	Organic matter (%)	Nitrate-Nitrogen (mg kg^−1^)	Electrical conductivity (dS m^−1^)	P_2_O_5_ (mg/100 g)	K_2_O (mg/100 g)	Rock content >2 mm (%)	Particle size distribution (%)		
									Sand (2–0.05 mm)	Silt (0.05–0.002 mm)	Clay (<0.002 mm)
0–10	7.77	0	0.892	6.03	0.060	1.02	10.2	0.42	68.6	21.9	9.5
10–20	7.33	0	0.806	7.89	0.024	0.81	7.8	1.21	71.8	19.0	9.2
20–30	7.20	0	0.813	7.40	0.026	1.06	6.7	0.76	71.5	19.2	9.3
30–40	7.17	0	0.880	7.82	0.025	0.68	5.5	1.55	72.1	18.7	9.2
40–50	7.26	0	0.842	6.41	0.025	1.77	5.5	1.36	72.5	17.4	10.1
50–60	7.18	0	0.801	6.69	0.028	0.77	5.5	1.02	71.9	17.6	10.5
60–70	7.23	0	0.824	8.17	0.033	1.60	5.5	1.08	71.8	18.3	9.9
70–80	7.17	0	0.734	6.69	0.035	1.48	5.5	1.19	71.8	18.4	9.8
80–90	7.30	0	0.512	7.50	0.074	1.27	5.5	1.26	76.2	13.2	10.6
90–100	8.04	0.95	0.248	7.47	0.073	1.10	5.5	0.18	77.7	12.1	10.2
0–100	7.37 (±0.09)	0.10 (±0.09)	0.735 (±0.06)	7.21 (±0.21)	0.040 (±0.006)	1.16 (±0.11)	6.3 (±0.47)	1.00 (±0.13)	72.6 (±0.8)	17.6 (±0.9)	9.8 (±0.16)

Data refer to two undisturbed plots outside the forest canopy up to a total depth of 1 m at 10 cm interval and for the whole soil depth. Numbers between brackets indicate the SE.

In regard to the particle size distribution, the rock content is around 1% with the lowest values in the upper (0–10 cm) and lower (90–100 cm) soil layers. Sand represents the majority with an average value of 72.6% increasing with the increase of the soil depth. Silt and clay represent, respectively, the 17.6 and 9.8% of the particle size distribution (Table 1).

**Figure 1:**
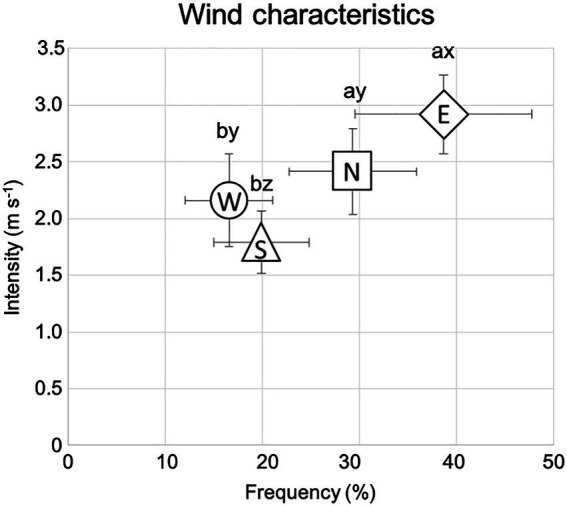
Wind data collected near the study area averaged on the growing season base (June to September), from 2012 to 2020, and according to the four cardinal directions (N-north, S-south, W-west, and E-east). Bars indicate the standard error of the mean. *a* and *b* indicate significant differences (*p* < 0.05) in wind frequency (%). *x*, *y*, and *z* indicate significant differences (*p* < 0.05) in wind intensity (m s^−1^).

The annual average temperature is 0.6 ± 0.45°C, while the summer average temperature is 16.29 ± 0.41°C. The mean air temperature of the warmest month (July) is 16°C, while that of the coldest month (January) is −22°C. The length of the growing season varies between 110 and 130 days. The average annual precipitation during the experiment (2000–2019) was 196 mm, according to the Lun soum weather station [The National Agency for Meteorology and Environmental Monitoring of Mongolia (NAMEM), 2019]. Precipitation usually occurs between June and August and accounts for 80–90% of the total annual rainfall. The mean annual potential evapotranspiration is 752 mm. There are two prevailing winds during the growing season (June–September), the first from the east and the second from the north, with the eastern wind being the dominant one (Figure 1). Winds from the south and the west represent the second group of prevailing winds with wind from the west being the dominant one (Figure 1; data 2012–2020 from the *Green Belt* project 47°52′46.13′N 105°19′17.14′E; LI-7500DS, LI-COR Biosciences, United States).

Vegetation is typical of the genuine dry bunchgrass steppe dominated by xerophytic and meso-xerophytic graminoids [e.g., *Stipa krylovii* Roshev., *Cleistogenes squarrosa* (Trin.), *Agropyron cristatum* (L.) Gaertn, and *Artemisia frigida* (Willd.), and, in degraded lands, *Artemisia adamsii* (Besser), *Carex duriuscula* C.A. Mey., and *Leymus chinensis* (Trin.); Ulziykhutag, 1989; Lavrenko et al., 1991].

### Plant material and management techniques

In May 2011, 2-year-old seedlings of *U. pumila* (grown from seeds) were acclimated in the open *Green Belt* project nursery and transplanted into 60-cm-deep holes with a diameter of 50 cm. At transplanting time, *U. pumila* seedlings were 51 ± 1.14 cm in height with a diameter at the root collar of 0.33 ± 0.01 cm. Immediately after transplanting, and for the following 30 days, all seedlings were irrigated with the same amount of water (40 L in total, 1.3 L day^−1^).

After seedling stabilization, water emitters were placed at a distance of 10 cm from the seedlings, and four different watering regimes were applied: no watering (control), 2 L h^−1^ = 0.25 mm m^−2^, 4 L h^−1^ = 0.5 mm m^−2^, and 8 L h^−1^ = 1 mm m^−2^. Seedlings were watered twice a week for the entire vegetative season (from the beginning of May to the end of August), with a duration of 5 h for each irrigation event. Watering interval and duration were based on the soil water infiltration rate analysis performed previous to the setting up of the experiment. In particular, the water field capacity was determined and its 50% was established as equal to the amount of water provided by the 8 L h^−1^ watering regime. During the entire experiment, the natural rainfall regime was over imposed on the watering regimes determining the actual amount of water available to the plants. Finally, watering was provided through CNL button drippers with different capacities of deliverable water. The irrigation hose system was connected to a water tank with a capacity of 50 m^3^. For each watering regime, two plots received a fertilizer treatment comprised of 500 g of solid granules of NPK or composted sheep manure (hereafter named compost). Fertilizers were mixed with natural soil before seedling transplantation.

In total, 12 plots were established four for each of the treatment combinations, which were watering along, watering plus NPK, and watering plus compost (hereafter named as management technique).

In detail:

- One plot for each of the four watering regimes, 32 seedlings each plot;
- One plot for each of the four watering regimes plus NPK, 16 seedlings each plot; and
- One plot for each of the four watering regimes plus compost, 16 seedlings each plot.

**Figure 2:**
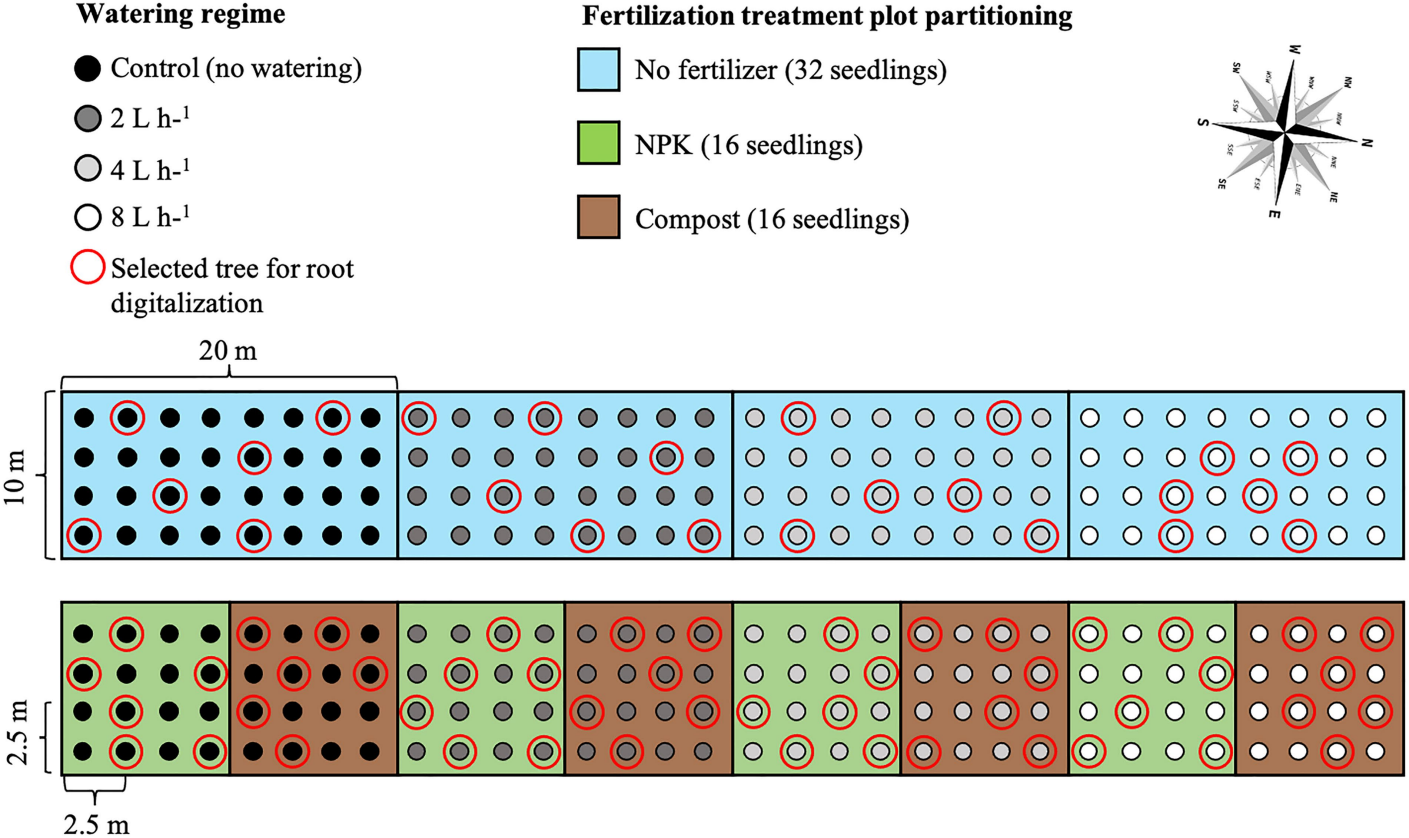
*Ulmus pumila* planting scheme of the afforestation site. The light blue rectangular area (20 m × 10 m) indicates the watering-only treatment (unfertilized trees), with 32 seedlings for each of the watering regimes. The green square areas (10 m × 10 m) indicate NPK fertilization, with 16 seedlings for each of the watering regimes. The brown square areas (10 m × 10 m) indicate compost fertilization, with 16 seedlings for each of the watering regimes. Black dots are relative to control trees (unwatered), dark gray dots indicate 2 L h^−1^ watered trees, light gray dots indicate 4 L h^−1^ watered trees, and white dots indicate 8 L h^−1^ watered trees. Red-encircled trees were those excavated for 3D digitalization.

Seedlings were planted in rows with a 2.5 m distance in between, and with a north–south orientation to ensure maximum light availability during the whole day (Johnson and Brandle, 2009; Figure 2).

### Root excavation and 3-dimensional architecture measurement

**Figure 3:**
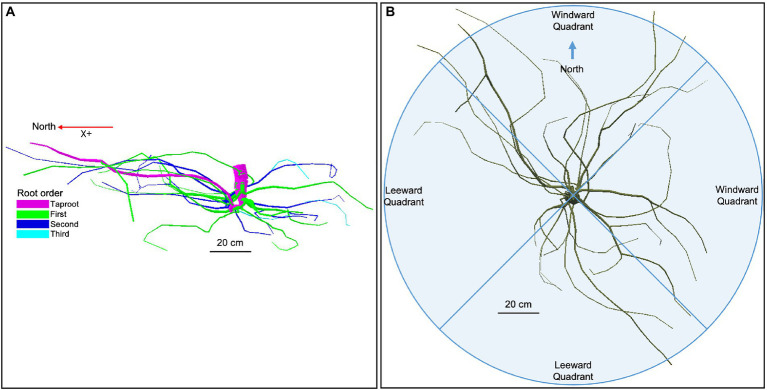
**(A)** Root hierarchy was digitized using the “acropetal-development approach,” which allows each root system to be reconstructed using the AMAPmod software for 3-dimensional analysis. In this image of an unfertilized tree which received a 2 L h^−1^ watering regime (ID 15)’, the taproot appears pink, first-order lateral roots emerging from the taproot are green, second-order roots are dark blue, and third-order roots are light blue. The X + axis is north-oriented. **(B)** Roots were excavated up to a distance of about 1 m from the trunk. Traits were assessed in four quadrants, two windward (east and north), and two leeward (west and south).

In mid-July 2019, 10 years after seedling transplantation, six *U. pumila* trees from each management technique were randomly selected (see red circles in Figure 2), cut at the root collar, and measured for height and diameter-at-breast height (DBH; see Byambadorj et al., 2021a). Of these six replicates for each treatment, we hand-excavated the root system up to approximately 0.8–1 m in depth and 1 m distance from the trunk. A single screw was driven into the bark at the root-stem interface to delineate the north. Two screws were partially drilled into the stump about 20 cm apart with their heads adjusted to horizontal level. Two more screws, perpendicular to the first two, were installed similarly. After cutting roots that were still attached to the soil, the root systems were carefully lifted from the soil and carried to an in-site field laboratory (Ger, a Mongolian traditional dwelling made by framed wood poles and felt cover) set up at the Lun soum nursery facility for analysis. We positioned the root systems hanging on wooden supports so that the exact inclination (achieved by adjusting the root so that the screw heads were at horizontal level) and north direction (positive X; Figure 3A) were restored.

The root system was discretized by a low magnetic field digitizer (Fastrak; Polhemus, Colchester, VT, United States) and encoded in a standard format (MTG) commonly used for representing branching topological relationships using AMAPmod software at different observation scales (Godin and Caraglio, 1998). Device characteristics (Danjon et al., 1999b) consisted of an electronic unit, a magnetic transmitter (Long Ranger; Polhemus), and a small hand-held receiver positioned at each point to be measured. The receiver measured the X, Y, and Z spatial coordinates within a sphere-wide electromagnetic field having a 4-m radius around the transmitter, which was sufficient for the root system sizes observed in this study. The transmitter was positioned approximately 1.5 m below and 2.5 m from the stump.

In the present work, the hierarchical branching structure (i.e., topology) was coded according to the “acropetal-development approach” (Danjon et al., 2013; Dumroese et al., 2019).

In particular, during the digitalization, the seed-origin radicle representing the primary roots axis (i.e., taproot) was designated as order zero (pink color in Figure 3A). The lateral roots emerging from the taproot were designated as first-order roots (green color in Figure 3A), the lateral roots emerging from the first-order roots were designated as second-order roots (dark blue color in Figure 3A), the lateral roots emerging from the second-order roots were designated as third-order roots (light blue color in Figure 3A), and so on (Zobel and Waisel, 2010).

Analysis was performed considering the first, second, and third-order only. The stump was determined subjectively as the portion of a taproot with a large diameter from where most of the large horizontal surface roots originated. The taproot was the largest vertical root originating directly from the stump. We started digitization at the root collar and followed a recursive path along the branching network as suggested by Danjon et al. (2005). Between branching points, intermediate measurements were performed to record changes in root direction and taper. A segment was defined as the root subdivision between two measured points. The average segment length was about 2 cm when roots were curved and approximately 10 cm when roots were straight. When a root cross-section was oblong, the largest diameter and its orientation, as well as the diameter perpendicular to the largest diameter, were recorded. All roots with a proximal diameter larger than 1 cm at the base were measured. Once the root system was digitized, it was dissected in different root diameter classes, independently of the spatial distribution. Roots were then oven-dried and weighed to obtain biomass values that were reported in a recent publication (Nyam-Osor et al., 2021). The output data file was analyzed using AMAPmod software (Godin et al., 1997), which handles topological structure at several scales, also providing a three-dimensional graphical reconstruction for data checking. Extracted data were exported to other software to perform specialized processing (see Statistical Analysis below). Root traits (i.e., total length, mean diameter, and mean branching density) were computed from three-dimensional digitized data of whole root systems. Root traits were considered as a function of depth and azimuth position, the west–east and north–south axes coinciding with the directions of the two prevailing winds. We chose two depths for assessment: 0–30 and >30 cm. Within each depth, we divided the space surrounding the taproot into four 90° quadrants: first windward (east), first leeward (west), second windward (north), and second leeward (south; Figure 3B).

### Statistical analysis

Mean wind frequency and speed were calculated from a 2012 to 2020 wind dataset, based on the growing season (June to September), and according to the four cardinal directions (N-north, S-south, W-west, and E-east). Two-independent sample *t*-tests were performed to evaluate significant differences among directions for both frequency and speed. A multivariate Generalized Linear Model (GLM) was employed to test the effect of the independent factors (i.e., predictors; soil depth, fertilization, watering regime, and cardinal direction) on root traits (i.e., dependent variables), such as total length, mean diameter, and branching density. When needed, the dependent variables were square root or log-transformed to ensure normal distributions and equal variances. Omega squared values (ω^2^) for each predictor were calculated to show the variation in the dependent variable attributable to the predictors. *Post-hoc* LSD tests were conducted to detect differences for the specific independent factor that resulted in significantly influencing the dependent variable. Analyses were applied to a 95% significance level. Statistical analysis was carried out using the statistical software package SPSS 25.0 (SPSS Inc., Chicago, IL, United States).

## Results

### Root length

The root length was significantly affected by soil depth (*p* < 0.001; 64.9% of the data variation), watering regime (*p* = 0.002; 4% of the data variation), and fertilization (*p* < 0.001; 4.3% of the data variation; Table 2). On the contrary, the cardinal direction did not influence the root length (*p* = 0.070; Table 2).

**Table 2:** Multivariate Generalized Linear Model (GLM) effect of independent factors (predictors: FER, fertilization treatments; WAT, water regime; DEP, soil depth; and QDR, quadrant) on dependent variables (root length, diameter, and branching density).

Dependent variable	Independent factor	*F*	*p*	*ω* ^2^
Length	FER	8.38	**<0.001**	0.043
	DEP	220.43	**<0.001**	0.643
	WAT	5.54	**0.002**	0.040
	QDR	2.44	0.070	0.013
				
Diameter	FER	3.59	**0.032**	0.040
	DEP	19.16	**<0.001**	0.141
	WAT	1.92	0.133	0.021
	QDR	3.92	**0.011**	0.068
				
Branching density	FER	4.28	**0.017**	0.031
	DEP	116.92	**<0.001**	0.544
	WAT	0.57	0.636	0.000
	QDR	0.51	0.676	0.000

*F* value (*F*), value of *p* (*p*), and omega square value are shown. Bold values indicate statistical significance (*p* < 0.05).

**Figure 4:**
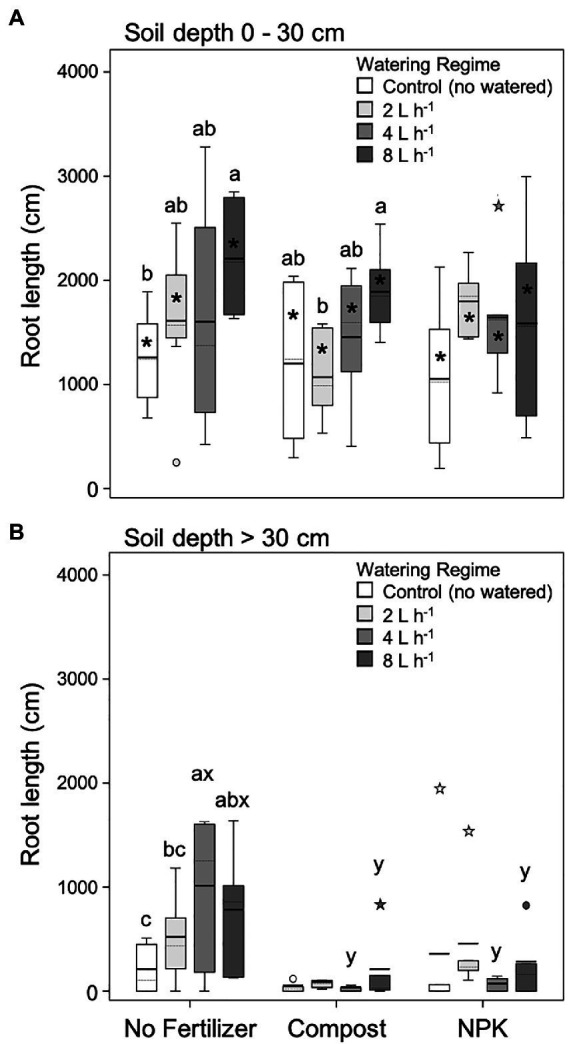
Root length at 0–30 cm **(A)** and >30 cm **(B)** soil depth for different watering regimes and fertilization treatments. Each depth was analyzed independently. Letters *a* and *b* indicate significant differences (*p* < 0.05) among watering regimes within each fertilization treatment; *x* and *y* indicate significant differences (*p* < 0.05) among fertilization treatments within each watering regime; the absence of letters reflects that no significant difference was detected. An asterisk (*) indicates a significant difference among soil depths within the same watering regime and fertilization treatment. Vertical boxes represent approximately 50% of the observations and lines extending from each box are the upper and lower 25% of the distribution. Within each box, the solid horizontal line indicates the mean value, while the dotted line represents the median (*n* = 6).

For both soil layers analyzed root (0–30 and > 30 cm) unfertilized trees’ root length increased with the increase of the watering regime (Figures 4A,B). In the upper soil layer (0–30 cm), the lowest and highest root length values were found in unwatered and 8 L h^−1^ trees, respectively (Figure 4A). At deeper soil depth (>30 cm), the lowest and highest root length values were found in unwatered and 4 L h^−1^, respectively (Figure 4B).

In the upper soil layer (0–30 cm), trees fertilized with compost showed a similar pattern to unfertilized trees that is the root length increase with the increasing watering regime, although significant differences were detected only between 2 and 8 L h^−1^ trees (Figure 4A). On the contrary, trees fertilized with NPK did not show differences among different watering regimes (Figure 4A).

At deeper soil depth (> 30 cm), in general, the root length significantly decreased (Figure 4B). In particular, fertilized plants with both compost and NPK did not show differences among different watering regimes, and, in the case of both 4 and 8 L h^−1^, root length was significantly lower than the unfertilized trees (Figure 4B).

### Root diameter

The root diameter was significantly affected by the soil depth (*p* < 0.001; 14.1% of the data variation), the fertilization (*p* = 0.032; 4.0% of the data variation), and the cardinal direction (*p* = 0.011; 6.8% of the data variation; Table 2). Watering regime did not influence the diameter of the root population (*p* = 0.133; Table 2).

**Figure 5:**
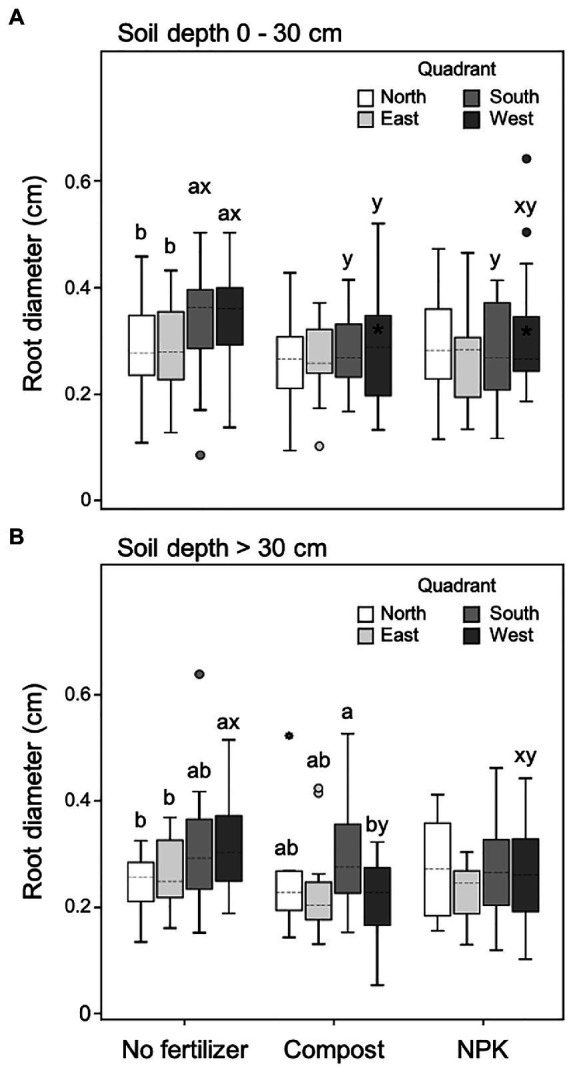
Root diameter at 0–30 cm **(A)** and >30 cm **(B)** soil depth for different cardinal quadrants and fertilization treatments. Each depth was analyzed independently. Letters *a* and *b* indicate significant differences (*p* < 0.05) among cardinal quadrants within each fertilization treatment; *x* and *y* indicate significant differences (*p* < 0.05) among fertilization treatments within each cardinal quadrant; the absence of letters reflects that no significant difference was detected. An asterisk (*) indicates a significant difference among soil depths within the same cardinal quadrant and fertilization treatment. Vertical boxes represent approximately 50% of the observations and lines extending from each box are the upper and lower 25% of the distribution. Within each box, the solid horizontal line indicates the mean value, while the dotted line represents the median (*n* = 24).

In the upper soil layer (0–30 cm), unfertilized trees showed significantly higher root diameter within both south and west quadrants (leeward) compared to the counter sectors north and east (windward). Moreover, in unfertilized trees, the root diameter of the leeward quadrants was also significantly higher than the diameter measured in the same quadrants of fertilized trees, independently of the fertilization type (Figure 5A). In particular, the root diameter of fertilized trees did not differ among the four quadrants (Figure 5A).

The root diameter did not change significantly from the upper to the lower soil layer with the only exception of the west quadrant of fertilized trees, which was higher in the upper soil layer (Figure 5A).

In the deeper soil depth (>30 cm) unfertilized trees had significantly higher root diameter in the west quadrant (leeward of the dominant wind), while the south quadrant showed intermediate values and the east and north quadrants the lowest (Figure 5B). In the case of trees fertilized with the compost, in the deeper soil depth, the root diameter was the highest and the lowest in the south and west quadrants, respectively (Figure 5B). On the contrary, trees fertilized with NPK did not show any difference in root diameter size among the four quadrants (Figure 5B). Also, with a deeper soil layer, unfertilized trees had significantly higher root diameter in the leeward sector of the dominant wind (east) than fertilized trees (Figure 5B).

### Root branching density

The branching density was significantly affected by the soil depth (*p* < 0.001; 54.4% of the data variation) and the fertilization (*p* = 0.017; 3.1% of the data variation; Table 2). Both watering regime (*p* = 0.636) and cardinal direction (0.676) were not influencing the root branching density (Table 2).

**Figure 6:**
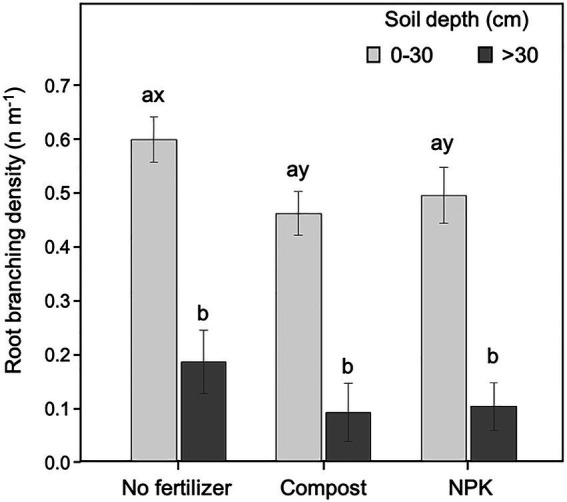
Root branching density at 0–30 and >30 cm soil depth for different fertilization treatments. Each depth was analyzed independently. Letters *a* and *b* indicate significant differences (*p* < 0.05) among soil depths within each fertilization treatment; *x* and *y* indicate significant differences (*p* < 0.05) among fertilization treatments within each soil depth. Each bar represents the mean (*n* = 24) ± 1SE.

The branching density was significantly higher in the upper soil layer (<30 cm) than at deeper soil depth (>30 cm) for both unfertilized and fertilized trees, independently of the fertilization type (Figure 6). Within the upper soil layer, the branching density in the unfertilized trees was significantly higher than in those fertilized (Figure 6). At the deeper soil layer, the pattern was similar (i.e., higher values of branching density in unfertilized trees than in fertilized) but differences were not statistically significant (Figure 6).

## Discussion

The spatial distribution of roots into the soil is of fundamental importance for a tree’s ability to both search for resources (Yang et al., 2014, 2017; Tan et al., 2021) and anchor to the soil (Dumroese et al., 2019; Saint Cast et al., 2019; Montagnoli et al., 2020). This ability is determined by the high degree of plasticity that characterizes the root system and that represents a compromise between optimum nutrient foraging and plant mechanical stability to face external mechanical forces such as prevailing winds. In the view of climate change intensifying meteorological events such as drought and windstorms (Brunner et al., 2015), optimal root architecture may represent a key factor for forest stability (Yang et al., 2017; Montagnoli et al., 2019; Freschet et al., 2021). This is especially true for afforestation projects that have been developed as a future solution for the (i) mitigation of climate change (Doelman et al., 2020), (ii) reduction of arid-lands degradation (Olsson et al., 2019), and (iii) creation of forest shelterbelts which through land-use changes might enhance the life quality of indigenous people (Wu et al., 2019). The setting up of new forest shelterbelts is dependent on the management technique that is adopted for optimal seedling establishment and growth. Thus, a full understanding of plant development concerning watering regimes and fertilization type is crucial for both guaranteeing the success of afforestation projects and the appropriate use of resources, which might be scarce, especially in arid environments.

In recent work (Nyam-Osor et al., 2021), was highlighted that in *U. pumila* (L.), the build-up of root biomass for most of the root classes based on diameter size positively correlated with increasing levels of watering, while the application of fertilizers led to root growth suppression. In the present work, how root length, diameter, and branching density of *U. pumila* are influenced by different management techniques and three-dimensionally organized with respect to rooting depth, and prevailing winds was investigated. The aim was to understand whether these techniques could have a direct influence on root architecture and, consequently, on tree stability through root anchorage mechanisms as well as on the ability to search for nutrients at deeper soil depths.

Findings related to root length in the presented manuscript are in line with previous results concerning the biomass (Nyam-Osor et al., 2021). Indeed, in the upper 30 cm of soil, the root length was positively correlated with watering regimes although this effect was strongly reduced by the application of fertilizers (compost and NPK). However, findings from other studies are controversial, since both root suppression (Wright et al., 2011) and proliferation (Farley and Fitter, 1999) have been reported to depend on different environmental conditions and the plant species considered (Cabal et al., 2021). In our case, the observed root length suppression was related to the interplay of different factors, such as fertilization type and the rate and time of its application as well as the sandy soil characterizing the study area (Nyam-Osor et al., 2021). In particular, was highlighted that the type and quantity of the fertilizers used to result in a large concentration of ammonium, organic acids, and urea-based fertilizer which can inhibit root growth (Zucconi et al., 1981; Chanyasak et al., 1983a,b; Wong, 1985; Chen et al., 2020). In the long term, ammonium immobilization by soil microorganisms may result in deficiency problems (Bengston and Cornette, 1973; Terman et al., 1973; Busby et al., 2007; Alburquerque et al., 2012; Abubaker et al., 2015). Moreover, characteristics of the applied manure were associated with an increased concentration of ammonium, which was related to the inhibition of primary root elongation, and root biomass (Tian et al., 2008; Giehl and von Wiren, 2014; Morris et al., 2017; Abubaker et al., 2020) and deep roots development (Comfort et al., 1988). A similar reduction in rooting depth was found by Svoboda and Haberle (2006) in winter wheat fertilized with a high rate of nitrogen (200 kg ha^−1^). Finally, the high N fertilizer application rates may have induced a growth priority of aboveground structures while repressing root growth (Dong et al., 2008; Rogato et al., 2010; Zhang et al., 2017). Therefore, if these fertilization inputs are considered to result in stress-related to resource availability, then a recent model (Cabal et al., 2021) predicts that plants respond to such stress by reducing both root range and root density.

Although root length measured in the deeper soil layer (>30 cm) showed the same pattern as that found for the upper soil layer, the values were dramatically lower. This result is not surprising since Jackson et al. (1997) showed that, considering the averaged root distribution for all ecosystems on a global scale, 75% is found in the top 40 cm of soil, while this proportion decreases rapidly at higher depths. However, unfertilized trees had significative higher values of root length than fertilized ones highlighting that the decrease in root length with increasing soil depth was of a higher magnitude in fertilized trees while watering regimes had no influence. Regarding this topic, Dupuy et al. (2005, 2007) widely remarked on how rooting depth influences the resistance to uproot through the increase of the soil/root cohesion as well as on how these mechanisms occurring during failure are a determinant parameter, especially in sandy-like soil. We therefore might speculate that the reduction of rooting depth occurring in our study site due to fertilization and characterized by sandy soils would lead to a future forest stand more susceptible to anchorage failure. In addition, papers in a recent topic collection reported that variations in rooting depth may be a key functional trait for determining plant survival and growth in drought-prone regions (Ji et al., 2021; Lihui et al., 2021; Montagnoli et al., 2022). The shallow root system as observed in our study could be more vulnerable to drought stress since the ability to capture water and nutrients at deeper soil layers would be quite reduced.

It was observed that our study site is characterized by two prevailing winds: one blowing from the east, with the highest intensity (i.e., dominant), and one blowing from the north, with the second-highest intensity. When root traits were analyzed in relation to the four wind-related quadrants corresponding to the windward and leeward sectors, further differences related to management techniques were unveiled. In particular, our results highlighted that in the two leeward quadrants (i.e., west and south), and for both soil layers analyzed (0–30 and > 30 cm) unfertilized trees developed roots with a larger diameter, which can be ascribable to root plastic strategies for enhancing tree anchorage in response to the double wind direction forces. Indeed, on the leeward side, the roots are subject to compressive and bending forces that are transferred to the soil (Stokes, 1994), so the stronger the soil and root-soil bond, the larger root surface area on the leeward side, and the higher the uprooting force that can be resisted (Stokes, 1994; Stokes et al., 1996). In particular, it has been widely demonstrated that roots can respond to prevailing winds by selectively increasing root number, diameter, length, and volume to enhance tree anchorage (Stokes et al., 1995; Nicoll and Ray, 1996; Danjon et al., 2005; Tamasi et al., 2005; Danquechin Dorval et al., 2016; Saint Cast et al., 2019). In particular, for the increase in root diameter observed in the present study, the mechanical enhancement of these roots is due to diameter-dependent characteristics, such as resistance to breakage, tensile maximum force, and stiffness which are generally expressed as a power-law function of the root diameter (Ziemer and Swanston, 1977; Coutts, 1987; Nilaweera and Nutalaya, 1999; Kondo et al., 2004; Genet et al., 2010; Vergani et al., 2012). Similar findings from other studies were related to the response of roots to environmental stimuli (Rellán-Álvarez et al., 2016) with particular attention to the mechanical forces due to both slope and wind (Danjon et al., 2005; Lombardi et al., 2017; Dumroese et al., 2019). However, Yang et al. (2014) pointed out that wind-induced mechanical forces act in the entire windward-leeward direction. Thus, it is reasonable to speculate that to obtain more effective support; the unfertilized *U. pumila* trees have developed larger leeward roots. Unfortunately, when fertilized trees were analyzed, the root diameter was not different among the four wind-oriented cardinal quadrants. These data further indicated a lack of root plasticity for fertilized trees specifically referring to the root response to the wind-originated mechanical forces.

Finally, in the present study, root branching density was dramatically reduced by the soil depth independently of the fertilization treatment. This decreases in branching density with the increase of soil depth has been often observed in different species and is related to both oxygen and nutrient availability gradients in the soil, which are higher in surface layers (Pagès et al., 2004). However, in our case, the root branching density in the upper soil layer was significantly lowered by the application of fertilizers. As for the other root traits discussed so far the observed reduction of root branching density may be related to both the rate and time of application as well as to the type of fertilizer used, which are important factors for meeting plant needs particularly in dry/arid lands (Abubaker et al., 2020). In a review study, Jacobs and Timmer (2005) highlighted that fertilization may dramatically alter rhizosphere chemical properties and electrical conductivity leading to the inhibition of the root system growth by reducing soil osmotic potential and creating specific ion toxicities. We may then infer that a general reduction of the surface branching density, independently of the cardinal quadrants, might be related to a slower dissipation of mechanical forces and, thus, may lead to weaker tree stability (Stokes and Mattheck, 1996).

Altogether these data fully support our hypothesis related to the suppression of the measured root traits by fertilization and a lack of root plasticity, which is especially relevant in deeper layers of the soil for the root length, in the surface layer for the branching density and the wind-oriented quadrants for the root diameter. We therefore might speculate that the reduction of rooting depth, root diameter, and branching density due to fertilization would lead to a future forest shelterbelt more vulnerable to mechanical constraints and, thus, more susceptible to anchorage failure. Also, the susceptibility to mechanical forces is likely to increase when the *U. pumila* trees will reach maturity and both the weight of above-ground organs and the wind force acting on the larger tree canopy will further increase the mechanical forces they are subjected to (James et al., 2006; Hale et al., 2012; Montagnoli et al., 2020; Sellier and Suzuki, 2020).

## Conclusion

Our findings highlight that in the upper 30 cm of the soil increasing the levels of watering directly increased the development of root length. However, this increment was significantly reduced when trees were fertilized, either with compost or NPK. Below 30 cm soil depth, a similar root developmental pattern was observed, although to a lesser extent. Fertilized trees showed a further reduction in root growth resulting in a significantly lower root length at high soil depth than unfertilized trees. Unfertilized trees showed clear root plasticity in adapting to the two prevailing winds (east and north), resulting in having in both soil layers high values of root diameter in the leeward quadrants. This peculiar radial growth pattern is known to enhance tree anchorage. In addition, in the upper soil layer, the root branching density of unfertilized trees was significantly higher than fertilized ones, independently of the watering regime and cardinal quadrants, another root plasticity strategy for enhancing tree anchorage in response to wind forces. Unfortunately, root development suppression by fertilizer application was reflected in the strong reduction of root plasticity in response to environmental cues with trees not having adopted the above-mentioned strategies to enhance plant anchorage. In conclusion, the fertilization of *U. pumila* trees used to afforest the semi-arid steppe of Mongolia significantly reduced the deepening of root development, the morphological and architectural adaptation to wind-generated mechanical forces, and the surface branching density. These effects would probably lead to a future forest shelterbelt more prone to drought and storms, events exacerbated by climate change.

## Data availability statement

The raw data supporting the conclusions of this article will be made available by the authors, without undue reservation.

## Author contributions

AM, BN-O, and DC conceived the research project and developed the study plan. BN-O, S-OB, DC, and AM were responsible for the field excavation. BN-O provided primary funding. AM and DC dealt with the methodological approach and the experimental design. AM performed the digitalization, was responsible for the data collection and interpretation, wrote the manuscript, and dealt with the revision process and finalization. BL performed the three-dimensional data extraction, arrangement, and analysis. AM and BL provided the 3D visualization. MT performed the data analysis and chart visualization. GSS and DC provided important insights into the research process. All authors contributed to the article and approved the submitted version.

## Funding

This work was funded by the Fellowship Grant (P2019-3635) by the National University of Mongolia and the Korea-Mongolia Joint *Green Belt* Plantation Project.

## Conflict of interest

The authors declare that the research was conducted in the absence of any commercial or financial relationships that could be construed as a potential conflict of interest.

## Publisher’s note

All claims expressed in this article are solely those of the authors and do not necessarily represent those of their affiliated organizations, or those of the publisher, the editors and the reviewers. Any product that may be evaluated in this article, or claim that may be made by its manufacturer, is not guaranteed or endorsed by the publisher.
